# Who’s learning? Using within-family studies to understand personalized learning

**DOI:** 10.1038/s41539-020-00082-4

**Published:** 2021-02-01

**Authors:** Sophie von Stumm, Jasmin Wertz

**Affiliations:** 1 Department of Education, University of York, York, UK; 2 Department of Psychology & Neuroscience, Duke University, Durham NC, USA

**Keywords:** Education, Psychology

Children differ in their abilities, skills, and interests. To deal with these differences productively in education, in ways that maximize the learning and development of every child, the idea of ‘personalized learning’ has been gaining popularity. Personalized learning seeks to optimize the fit between instruction and learner^1^ (cf. person–environment fit^2^), for example, through implementing individualized, flexible learning schedules; matching students to difficulty levels of instruction according to their learning abilities (i.e., streaming or tracking^3^); and encouraging student choice in education.

Although typically thought of in the context of classrooms and schools, children’s first experience of personalized learning occurs in the family home. Parents deal with their children’s heterogeneity in abilities, skills, and interests by altering their behavior, and even the home environment, to meet their children’s individual characteristics and needs^4–6^. For example, parents might read more often to a child who appears to be interested in books, or take this child to the public library, while they might indulge a child who’s a gifted swimmer with a season ticket to the local pool^4^. Here we propose that studying how parents tailor family environments to their children’s characteristics and needs can provide insights into personalized learning that are also applicable to classroom contexts. We refer to such studies as within-family designs, because they include data from at least one parent and two or more of their children. Data on children’s characteristics may include children’s cognitive abilities, personality, genetic dispositions, and other learning-related traits that can differ between children growing up in the same family. The purpose of this commentary is to describe how within-family studies could be used to test hypotheses about personalized learning. Specifically, we discuss three key questions about personalized learning that within-family studies can help answer, including (1) how the correspondence between learner characteristics and learning environment influences learning outcomes (i.e., optimal match), (2) which contextual and instructor characteristics encourage or hinder the process of personalizing learning, and (3) what the implications of personalized learning are for achievement inequalities between children.

## Optimal match between learner and environment

The concept that some learning environments are more or less effective for particular individuals, depending on their learning abilities and needs, is known as ‘aptitude–treatment interaction’ or ‘ATI paradigm’^7,8^. The ‘treatment’ term refers to the environment available to the learner, for example, instructional strategies or learning materials, whereas ‘aptitude’ refers to those individual differences or learner characteristics that forecast an individual’s probability of success under a given treatment^7–11^. Although aptitude–treatment interactions are thought to be common in all formal and informal educational settings, they have not been conclusively demonstrated^7^. Testing aptitude–treatment effects in classrooms entails assessing how various degrees of (mis)match between instruction and learner characteristics affect learning outcomes. Such study designs are complex and require extremely large sample sizes to achieve sufficient statistical power, especially because effect sizes are likely to be small^10–12^.

Within-family designs can potentially afford greater statistical power than conventional classroom- and school-based studies for testing the effects of different degrees of (mis)match, because collecting data from a large sample of family dyads with two siblings and a parent is more feasible than assessing the same number of classrooms, each of which typically enroll more than one teacher and two students. Within-family designs enable testing whether a child, who is more or less similar to their parent in learning-associated traits compared to their sibling, will achieve more or less learning, across families that differ in the degrees of (mis)match within their dyads. The rationale is that parents’ learning-associated traits will inform the home environment and parenting that they provide for their children^4–6^. The question is then: of two or more children in a family, will the one child learn best whose learning-related traits are most closely matched to the learning-related characteristics of their parents and their home environment? This question is distinct from testing gene–environment correlations that occur when the exposure to environmental conditions (i.e., the family home) depends on an individual’s genotype and that are differentiated into active, passive, and evocative^4^. Instead, we ask to what extent the (mis)match between environment and child characteristics, which are influenced by both genetic and environmental factors, predicts children’s differences in learning outcomes.

We are not aware of a prior empirical study that modeled the extent to which the alignment between children’s characteristics and their environments influenced their learning outcomes. A within-family study could, for example, test the hypothesis that the sibling, whose cognitive ability resembled that of their parents more closely, performed better in school, because parents may create an environment that is matched better to this child’s proclivities than to those of the other sibling. Such matching effects may make a significant contribution to children’s development, even though they are likely to be smaller than the main effects of child ability, akin to findings from the person–environment fit literature^2^. Through comparisons across families, we could also test if there was an ‘optimal’ match between a child’s cognitive abilities and their parents’ for improving learning outcomes, for example, if children’s learning was enhanced if their cognitive abilities differed slightly from their parents’ rather than being perfectly matched to them. When translating these findings to the context of classrooms and schools, the focus would be on the match between the characteristics of learner and instruction, such as pace, style, and complexity, rather than the teacher’s traits.

## Contextual and instructor characteristics that shape personalized learning

Personalized learning involves active participation from children and instructors. On the children’s side, personalization occurs because they select themselves into environments, such as peer groups at school^13^, and evoke responses from their social context, for example, their teachers^14^. Optimal learning environments recognize and encourage learners’ active participation^15^, but the process of how ‘making one’s own environment’^12^ plays out in educational settings is poorly understood. Within-family studies can help here by elucidating how contextual characteristics, such as availability of resources in the home, affect the extent to which children personalize their home environment and evoke differential reactions from their parents.

On the instructors’ side, personalization occurs because instructors differ in their willingness and capacity to personalize their students’ learning^16^. Identifying instructors’ characteristics, such as experience, attitudes, and instruction style, and relevant contextual factors, such as school and classroom resources that influence how instructors respond to students’ heterogeneity, is key to finding ways for improving instructors’ effectiveness in personalizing learning^17^. Within-family studies can help here by identifying the factors that affect parents’ personalizing of their children’s environments^18^, which are likely to also apply to teachers. For example, two or more siblings will differ in their passion for reading; yet, some parents will buy them the same number of books regardless, while others may offer alternative gifts to the less reading-enthusiastic siblings. Within-family designs enable testing to what extent parents’ responsiveness may be driven by family-level characteristics (e.g., socioeconomic status) or by differences in their children’s traits, and if parents’ differential responsiveness occurs for some but not other trait domains. These mechanisms are different from gene–environment correlations, which imply that home environments and parenting are, on average, aligned with children’s characteristics^4^. Instead, the guiding question is: why are some parents and by extension, the rearing environments that they provide for their children, more attuned to their children’s needs than others?

## Personalized learning and achievement inequalities

Personalized learning has been hailed as an approach to reduce educational inequities^19^. However, some methods of personalizing education, for example, ability tracking or streaming, might increase achievement gaps and educational inequality^3^, although the empirical evidence for this is mixed^20^. Within-family designs can help address the possibility that personalizing learning may have negative consequences for some learners, by testing whether parents’ differential treatment of their children is associated with an increasing divergence in children’s learning outcomes over time^21^. For example, the trait differences of siblings, who in response to their heterogeneity receive differential treatment from their parents, may magnify over time, while differences among siblings who are treated just the same, regardless of their individual characteristics, may increase less or even remain stable. In other words, we suspect that some parents will, perhaps inadvertently, augment the observable differences between their children, while others will establish parenting strategies and rearing environments that decrease siblings’ differences. Within-family designs are not suitable for testing if socioeconomic background or ethnicity increase or reduce siblings’ differences in learning outcomes over time, because these characteristics are shared among children in the same family. Yet, within-family designs can be applied to test changes in the magnitude of children’s differences in their individual characteristics, such as in their interests or cognitive abilities. Understanding the possible consequences of personalized learning is pivotal for educators and policymakers to ensure that children receive equally fair education opportunities.

## Conclusion

Educators, researchers, and policymakers are concerned with the question of how learning environments can be designed to best meet the needs of children who differ in their abilities, skills, and interests. In this article, we have outlined how studying differences in learning experiences among children within their families can provide insights about the optimal ‘match’ between learner and environment; about the characteristics of learners and instructors that promote or hinder personalized learning; and about the implications of personalizing learning for educational inequalities between students. We propose that within-family designs are a useful addition to the educational researcher’s toolbox for studying personalized learning.
